# Computational Modeling of the Mechanism of Urease

**DOI:** 10.1155/2010/364891

**Published:** 2010-09-20

**Authors:** Håkan Carlsson, Ebbe Nordlander

**Affiliations:** Chemical Physics, Center for Chemistry and Chemical Engineering, Lund University, Box 124, 221 00 Lund, Sweden

## Abstract

In order to elucidate aspects of the mechanism of the hydrolytic enzyme urease, theoretical calculations were undertaken on a model of the active site, using density functional theory. The bridging oxygen donor that has been found in the crystal structures was determined to be a hydroxide ion. The initial coordination of urea at the active site occurs most likely through the urea oxygen to the nickel ion with the lowest coordination number. This coordination can be made without much gain in energy. The calculations also showed that weak coordination of one of the urea amine nitrogen atoms to the second nickel atom is energetically feasible. Furthermore, a proposed mechanism including a tetrahedral intermediate generated by hydrolytic attack on the urea carbon by the bridging hydroxide was modeled, and the tetrahedral intermediate was found to be energetically unfavorable relative to terminal coordination of the substrate (urea).

## 1. Introduction

The hydrolytic enzyme urease is responsible for the catalytic decomposition of urea to volatile ammonia and carbon dioxide [1]. The enzyme releases ammonia and carbamate, which in turn spontaneously generate the products. The enzyme was studied early [2] and has generated interest for several reasons. It has been suggested to play a role in bacteria-induced ulcers [3, 4], and its activity has also been found to have implications in agriculture through the volatilization of urea—a commonly used fertilizer—that is generated by the enzyme [5]. Urease was also the first enzyme to be found to be dependent on nickel for its function [6], which has made it an interesting target site for bioinorganic model chemists [7]. 

**Figure 1:**
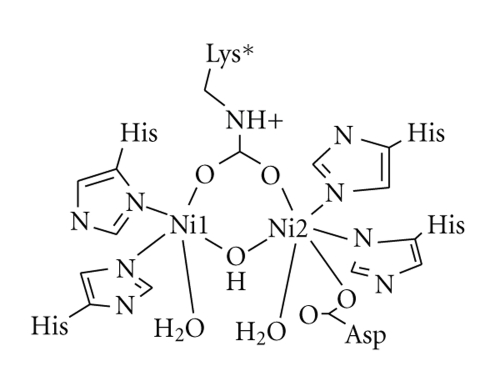
Schematic depiction of the structure of the active site of *Bacillus pasteurii* urease [11].

The protein structure of urease from *Klebsiella aerogenes* was first solved in 1995 in [8], and since then several other structures of the enzyme, with or without bound inhibitors have been determined [9–12], including structures from *Bacillus pasteurii *[10–12] and *Helicobacter pylori* [13]. The active site contains two nickel ions with an interatomic distance of about 3.5 Å (Figure 1). The ions are bridged by a carbamylated lysine and an oxygen donor. In addition to the bridges, one of the nickel ions (Ni1) is coordinated by two histidines and a water molecule. The coordination of Ni2 is similar to the one of Ni1 and includes two histidine residues, a water molecule and a terminally bound aspartate. 

**Figure 2:**
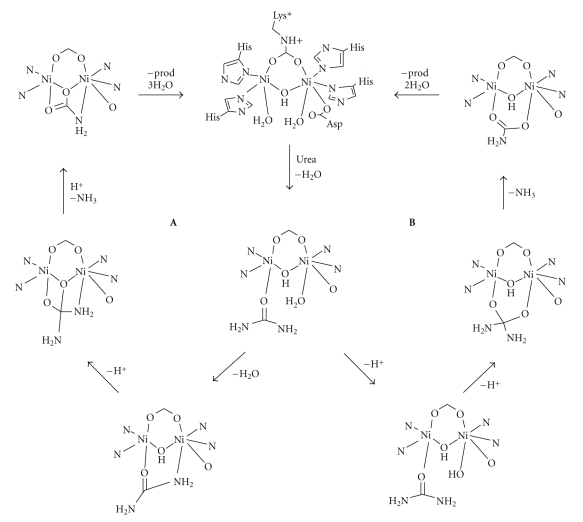
Schematic depiction of two proposed mechanisms for urease, **A** [11] and **B** [15].

A number of proposals have been made regarding possible reaction mechanisms. Consensus has been reached regarding the initial coordination of urea to the active site, which has been suggested to occur through the urea oxygen attacking the vacant coordination site on Ni1 (Figure 2), but there are divergent proposals regarding the subsequent steps. The initially suggested mechanism involves the attack on the urea carbon by a hydroxide that is terminally bound to Ni2. This leads to an intermediate that bridges the two metals and can release ammonia to form products [14, 15]. Based on the structure of an inhibitor complex, Benini et al. [12, 16] suggested a mechanism involving a secondary coordination of one of the urea nitrogen atoms to Ni2, which positions the substrate for an attack from the bridging hydroxide (water). The bridging coordination of the urea substrate was supported by Pearson et al. [17], but these investigators have suggested that the nucleophile is not the bridging hydroxide, but rather a water/hydroxide coordinated to Ni2.

 A few computational studies on the mechanism of urease have been published during the past decade. These include molecular mechanics studies by Zimmer [18, 19] and Smyj [20] and DFT studies by Merz et al. [21, 22]. The latter density functional study was carried out at the B3LYP level and modeled the entire direct coordination sphere of the dinickel site with the coordinated amino acid residues truncated so that the full functional group (imidazole (His), carbamate (carbamylated Lys) and carboxylate (Asp)) were represented. This study aimed to discern between the two proposed mechanisms that involve a bridging substrate [16, 17], but it also evaluated the original suggestion that urea is terminally coordinated. For the mechanism involving a bridging intermediate, the density functional study indicated that the bridging hydroxide is the actual nucleophile, but it was found that the rate determining transition states of these mechanisms and that involving a terminally coordinated urea molecule were very similar and that it was not possible to discriminate between these mechanisms on the basis of the calculations. In more recent molecular dynamics and quantum mechanical simulations, Estiu and Merz [23, 24] have detected initial coordination modes of urea that involve either terminal coordination to Ni1 or a bridging coordination with coordination of the urea carbonyl moiety to Ni1 and simultaneous hydrogen-bonding interaction with one of the amine nitrogens with the bridging hydroxide, depending on the protonation state of surrounding amino acid residues.

 In this paper, we wish to present DFT-based calculations that have been carried out in order to study the urease mechanism. The structure of the active site after initial urea coordination has been modeled. The theory involving an attack by the bridging oxygen donor has been tested. Activation data from the literature, which list energies of activation to circa 50 kJ/mol [25, 26] were compared with the energy differences between the starting structure and identified intermediates.

## 2. Results and Discussion

Because of the computational expense of the DFT method, a survey was done to find the smallest dependable model on which to base the rest of the study. Three model systems were investigated. The smallest system contained only the atoms within three to four bonds from the metal ions. The second contained all atoms of the coordinating amino acid residues up to and including the *α*-carbons. The third model system also included some nonbonded residues in close vicinity. As starting structure, the native urease crystal structure from *Bacillus pasteurii* (PDB code 2UBP) was used [11]. In the model survey, the structure from the protein was extracted and then geometrically minimized. A possible intermediate structure with urea bond to Ni1 was also studied. The result showed that all the structures gave very similar results, both geometrically and in the difference of energy between the two studied models. This result indicated that the smallest of the models was suitable for the study. In the chosen model, only metal-bound ligands were included. The histidine residues were modeled as imidazoles, the aspartate residue was capped as a methyl group at the *β*-carbon while the carbamylated lysine was terminated similarly at the *ε*-carbon (Figure 1). The remaining ligands were left unchanged.

### 2.1. Determining the Resting Structure

Since the information provided by X-ray crystallography, especially protein crystallography, may be insufficient to accurately determine the presence and location of hydrogen atoms, different depictions of the nature of the bridging oxygen donor have been published [28–30]. As a part of preparing a suitable starting structure for the study, three different bridging ligands were considered, namely, O^2-^ (**1a**), OH^−^ (**1**), and H_2_O (**1b**). In order to avoid hydrogen bonding to the bridging ligand, which could by itself drastically change the energy of the structure, the aspartate ligand was turned away from the nickel center by rotating it 135° counterclockwise around the Ni–O bond. To allow comparison between the different structures throughout the study, the energy of the substrates (urea and water) and further on the intermediate compounds were added to the resulting energy of each calculated structure. In calculating the contributing energies, the energy of a “free” proton was needed. Due to the many histidines conveniently located around the active site, the energy of a proton was determined by the energy difference of a protonated histidine and a neutral histidine, which were both calculated independent of the urease model. The result from the calculations can be seen in Table 1. In the comparison of the different possible oxygen donors, the structure with the lowest energy (by a comfortable margin) was the hydroxide-bridged complex (structure **1**). The energies of structure **1a** and **1b** were 101 and 33 kJ/mol higher than for structure **1**, respectively. The identity of the bridging hydroxide ligand is in agreement with the computational results obtained by Suárez et al. [21]. If the bridging ligand was water and the aspartate side chain was allowed to point towards the bridging ligand, a proton transfer from the water to the aspartate occurred during the geometry optimization. This observed proton transfer is an indirect confirmation of the instability of the model complex **1b**. The overall structure of the energetically favoured model complex **1** is very similar to the published X-ray structure. The interatomic distance is slightly shorter (3.48 versus 3.6 Å in 2UBP), and the site also differs in the orientation of the rotated aspartate.

### 2.2. Coordination of Urea

**Figure 3:**
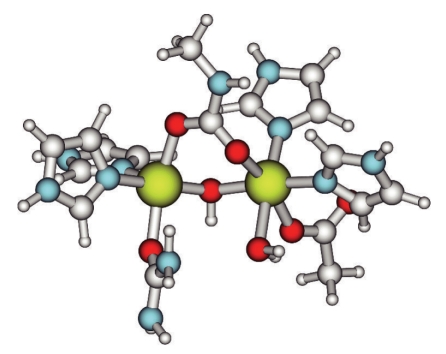
Structure of complex **2**—depicting the initial coordination of urea. The program Molden (see [27]) was used to generate the graphics.

In order to determine the mode of interaction as urea coordinates to the active site of urease, a docking experiment between the optimized model complex **1** and urea was carried out. A series of optimizations were performed moving urea from a distant position outside the active site and stepwise closer until a binding interaction was attained. Initially, urea was placed in the plane of the nickel ions and the carbamylate oxygen atoms, approximately 5.5 Å from the site. In order not to favor coordination to a specific nickel, the distance between the carbamylate carbon atom and the urea oxygen was chosen as the progressive distance. In the first optimization, this distance was 8 Å. After a number of iterations, which did not always result in the most favorable structure, the optimization was stopped, and the distance was shortened. This process was carried out repeatedly until urea was bound. The qualitative result was that urea stayed in a relatively centered position during the approach but as it came closer to the active site, it migrated towards Ni1, where it finally settled in a binding interaction with nickel. The urea coordinated through its oxygen atom in *trans* position to the carbamylated lysine on Ni1. Another effect of the stepwise optimization was that the water-Ni1 bond distance increased until finally water was released. The water molecule was omitted from the structure, which was reoptimized to structure **2** (Figure 3). The final urea to nickel distance is 2.12 Å. Compared to structure **1**, the internuclear distance is approximately the same (3.47 versus 3.48 Å). No major rearrangements are in other words needed in the binding of urea.

To test the viability of complex **2** as the first bound interaction between the urease active site and the substrate, some other possible structures were tested. The first one was coordination by urea *trans* to one of the histidines on Ni1, replacing the bound water (structure **2a**). A second alternative model was achieved by replacing the water molecule on Ni2 by coordinated urea (structure **2b**). The resulting energies are shown in Table 2. The two alternative structures are both disfavored by more than 30 kJ/mol relative to **2**; this is a significant energy difference comparable to ΔH^‡^ for the whole reaction.

### 2.3. Tetrahedral Intermediate

**Figure 4:**
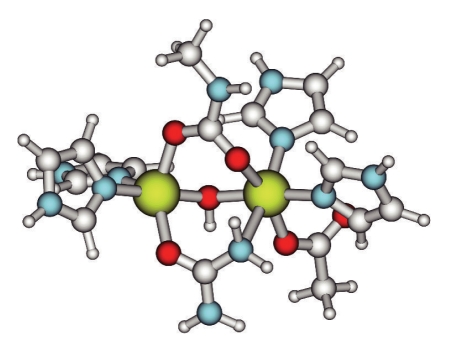
Structure of complex **3**. One of the urea nitrogen atoms is coordinated to Ni2, thus replacing the water molecule on Ni2.

In order to assess the possibility of a tetrahedral intermediate in the catalytic reaction of urease [16], a number of probable intermediates along the proposed pathway were studied. The choice of intermediates was based on what could be gathered from the suggested pathway and chemical intuition. Starting from **2**, the search for the most favorable configuration for complex **3**, in which one of the urea nitrogen atoms coordinates to Ni2, was done using a similar approach as in the two earlier cases. The distance between the closest urea nitrogen atom and Ni2 was fixed and shortened stepwise. During the approach, the Ni2-water distance grew longer until the water molecule was finally omitted and the structure with Ni2 and one of the urea nitrogen atoms within binding distance was optimized. The optimized structure for complex **3 **is shown in Figure 4. One of the nitrogen atoms has approached Ni2 to a bonding interaction. This replaces the water molecule on Ni2.

**Figure 5:**
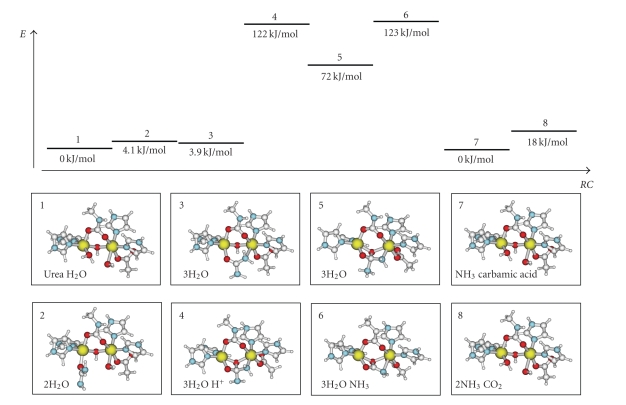
A reaction diagram showing the calculated energies of the intermediates found in the computational simulation of the urease reaction with the structures and added small molecules in boxes below (see text for detailed description of the intermediates).

 Starting from **3**, a nucleophilic attack on the bridging substrate was modeled by shortening the distance between the urea carbon atom and the bridging oxygen (Figure 5). The direct approach was again determined by stepwise optimizations. During the approach, the O–H bond in the bridging hydroxyl group was lengthened, and the proton was removed in the final optimized structure (**4**). Complex **5** was obtained by protonating the uncoordinated urea nitrogen atom in order to facilitate the release of ammonia. This lengthened the carbon nitrogen distance, but the intermediate was kept without completely removing the ammonia molecule. In complex **6**, involving the dissociation of ammonia to yield carbamate, the ammonia was completely removed (deleted) from the structure, and the structure of the resultant complex was optimized. The remaining structures were based on the resting state, **1**. The differences between complexes **1**, **7,** and **8** are the molecules that are added to get the comparable energy. In complex **7**, which involves the dissociation of carbamate, which gets protonated, and recoordination of water, the released carbamic acid and the previously released ammonia are added in order to obtain a comparable energy. The released carbamic acid is then assumed to spontaneously break down to carbon dioxide and ammonia, which are added in the optimization of complex **8**.

 The energies of the proposed intermediates and internuclear distances are summarized in Table 3. A graphical representation of the modelled reaction pathway is shown in Figure 5. There was a moderate increase in energy of about 40 kJ/mol before and after binding urea. Rather surprisingly, the formation of the ensuing coordination of one of the nitrogen atoms to Ni2 (structure **3**) did not lead to any larger energy change. The bridging coordination of urea was only disfavored over the terminally bound state by approximately 0.9 kJ/mol. However, it should be noted that the transition state is not studied and may be significantly higher. The Ni–Ni distance is again very similar, 3.47 Å. The effect on the urea molecule was also rather small. The C–N distance was 1.37 Å for the nitrogen coordinated to nickel, while the free nitrogen carbon bond was found to be approximately 1.35 Å. The distances in **2** were both about 1.36 Å. The geometry around the coordinated nitrogen had also shifted from a nearly planar structure, which is typical for urea, to a clear sp^3^ hybridized configuration. The optimized Ni–N(urea) distance is rather long (2.51 Å), suggesting a weak interaction. The Ni1–O(urea) bond distance was more or less unchanged.

**Figure 6:**
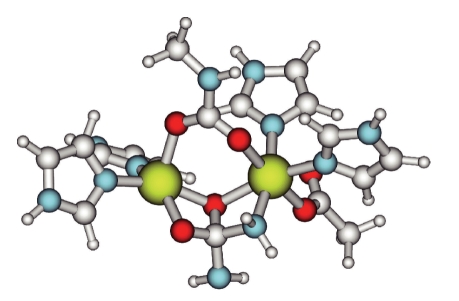
Structure of complex **4**—the tetrahedral intermediate. The bridging hydroxide and the urea have moved closer together so that a bond is formed. This was found to release the proton associated with the hydroxide bridge.

 The formation of **4** was found to require a fair amount of energy, as a large movement within the dinuclear site was needed to form the tetrahedral intermediate. The bridging oxygen donor between the two metals moved away from the nickel atoms and was positioned within bonding distance to the urea carbon. The Ni–O(phenolate) distance in structure **3** was 2.01 (Ni1) and 2.04 (Ni2) Å and in structure **4**, 2.07 (Ni1) and 2.16 Å (Ni2), respectively. In **4**, the partial double bond character of the C–N bonds in urea has disappeared and the hybridization of the carbon is clearly sp^3^. The C–N bonds are longer, 1.46 and 1.51 (see Figure 6) versus 1.37 and 1.35 Å in structure **3**. The nickel atoms have moved away from each other, and the internuclear distance is now 3.66 Å. The coordination of the bridging hydroxide has pulled urea closer to the dinuclear site, and the bonded Ni–N(urea) distance is shortened from 2.51 Å in **3** to 2.09 Å in **4**. The energy difference to go from structure **3** to **4** has been calculated to 111 kJ/mol. Considering that a transition state will lie higher in energy, it may be argued that the modeled reaction pathway is not likely to be a part of the mechanism of urease, which was earlier found to have an activation energy of 50 kJ/mol.

 The protonation of the uncoordinated urea nitrogen atom, to start the dissociation of ammonia, relieved some of the energy in structure **4**. The energy for the resultant structure **5 **is about 11 kJ/mol lower than in the previous structure. The protonated nitrogen moves away from the product carbon and was in the optimized state 1.70 Å from the carbon, which is about 0.20 Å further than in structure **4**. The geometry around the carbon has again started to become more planar. This has led to a strain that is relayed to the nickel ions, which have moved even further apart. The Ni–Ni distance was determined to 3.80 Å, which is a fairly long distance for this site. The move towards a completely planar product continued in structure **6**, in which ammonia was formally removed. This again pushes the nickel ions apart to 3.92 Å and adds energy to the structure. The energy for structure **6** is 142 kJ/mol higher than in the resting state. This energy is relieved in complex **7** and **8** when the product is released and water is recoordinated.

 As seen in Figure 5, the energy needed for the suggested pathway exceeds the empirically determined activation energy of 50 kJ/mol. Even if one assumes a large uncertainty in the calculated energies, they indicate that the tetrahedral intermediate is not a part of the urease mechanism. The activation energy for the calculated mechanism is expected to exceed 150 kJ/mol.

## 3. Conclusions

The binding and hydrolysis of urea at the active site of urease has been modeled. The resting state of the enzyme (active site) has been calculated. The calculations indicate that the bridging oxygen donor is a hydroxide ion; this structure is 101 and 32.9 kJ/mol more stable than the corresponding oxo- or water complexes. In agreement with previous inorganic model studies [31, 32] and proposed mechanisms [15–17, 21], the calculation further suggested that urea initially binds through its oxygen atom to Ni1 in the active site. The coordination was directed to the *trans* position relative to the carbamylated lysine, and the water ligand of the resting state was released.

A study of the proposed mechanism involving a tetrahedral intermediate based on the bridging hydroxy group (mechanism **A**, Figure 2) was carried out. In the study, four additional possible intermediates were studied. It was found that it is energetically possible to coordinate one of the urea nitrogen atoms to Ni2, but further transformation including the formation of a tetrahedral intermediate based on the bridging hydroxyl group is energetically unfavourable. Published empirical data gives activation energies of about 50 kJ/mol while the calculations indicate that 150 kJ/mol would be needed to reach the tetrahedral intermediate. Further studies involving computational modelling of the alternative mechanism involving nucleophilic attack by a terminally bound hydroxide on terminally bound urea (mechanism **B**, Figure 2) will be undertaken, and detection of transition states for the different mechanisms will be investigated.

## Figures and Tables

**Table 1:** Energy results from the calculation of the resting structure.

Structure	Calculated energy (Hartree)	Added small molecules	Adjusted energy (Hartree)	Relative energy (kJ/mol)
**1** (OH)	−13.6629	Urea and water	−15.9521	0
**1a** (O)	−13.6446	Urea, water and H^+^	−15.9134	101.
**1b** (H_2_O)	−13.6300	Urea, water and −H^+^	−15.9395	32.9

**Table 2:** Energy results from the calculation of the first coordination of urea.

Structure	Calculated energy (Hartree)	Added small molecules	Adjusted energy (Hartree)	Relative energy (kJ/mol)
**2** (Ni1 *trans*)	−14.8816	2 water	−15.9376	0
**2a** (Ni1 *cis*)	−14.3368	3 water	−15.9209	82.0
**2b** (Ni2)	−14.8821	2 water	−15.9381	36.7

**Table 3:** Energy results from the calculation of the mechanism involving a tetrahedral intermediate (mechanism A, Figure 2).

Structure	Calculated energy (Hartree)	Added small molecules	Adjusted energy (Hartree)	Relative energy (kJ/mol)
**1**	−13.6629	urea and water	− 15.9521	0
**2**	−14.8816	2 water	−15.9376	38.0
**3**	−14.3532	3 water	−15.9373	38.9
**4**	−14.3315	3 water and one H^+^	−15.8951	150
**5**	−14.3150	3 water	−15.8991	139
**6**	−13.6828	ammonia and 3 water	−15.8981	142
**7**	−13.6629	ammonia and carbamic acid	−15.9505	−33.9
**8**	−13.6629	2 ammonia and carbon dioxide	−15.9451	18.3
